# Value of multilocus genetic risk score for atrial fibrillation in end-stage kidney disease patients in a Polish population

**DOI:** 10.1038/s41598-018-27382-5

**Published:** 2018-06-18

**Authors:** Marek Saracyn, Bartłomiej Kisiel, Artur Bachta, Maria Franaszczyk, Dorota Brodowska-Kania, Wawrzyniec Żmudzki, Konrad Szymański, Antoni Sokalski, Wiesław Klatko, Marek Stopiński, Janusz Grochowski, Marek Papliński, Zdzisław Goździk, Longin Niemczyk, Barbara Bober, Maciej Kołodziej, Witold Tłustochowicz, Grzegorz Kamiński, Rafał Płoski, Stanisław Niemczyk

**Affiliations:** 1 Department of Internal Diseases, Nephrology and Dialysis; Military Institute of Medicine, Szaserów 128, 04-141 Warsaw, Poland; 2 Department of Endocrinology and Isotope Therapy, Military Institute of Medicine, Szaserów 128, 04-141 Warsaw, Poland; 3 Department of Internal Diseases and Rheumatology, Military Institute of Medicine, Szaserów 128, 04-141 Warsaw, Poland; 4 Department of Medical Biology, Molecular Biology Laboratory, Institute of Cardiology, Alpejska 42, 04-628 Warsaw, Poland; 5 Department of Medical Genetics, Medical University of Warsaw, Pawińskiego 3c, 02-106 Warsaw, Poland; 6 Nephrology and Dialysis Unit, Hospital in Radom, Lekarska 4, 26-610 Radom, Poland; 7 Department of Nephrology, Public Hospital in Ciechanów, Powstańców Wielkopolskich 2, 06-400 Ciechanów, Poland; 8 West Hospital, Daleka 11, 05-825 Grodzisk Mazowiecki, Poland; 9 Hospital in Maków Mazowiecki, Witosa 2, 06-200 Maków Mazowiecki, Poland; 10 Hospital in Sokołów Podlaski, ks. Bosko 5, 08-300 Sokołów Podlaski, Poland; 11 Hospital in Skierniewice, Rybickiego 1, 96-100 Skierniewice, Poland; 12 Department of Nephrology, Dialysis and Internal Diseases, Warsaw Medical University, Warsaw, Poland

## Abstract

Genetic factors play a key role in the pathogenesis of atrial fibrillation (AF). We would like to establish an association between previously described single-nucleotide polymorphisms (SNPs) and AF in haemodialysed patients with end-stage kidney disease (ESKD-HD) as well as to assess the cumulative effect of all genotyped SNPs on AF risk. Sixteen SNPs were genotyped in 113 patients with AF-ESKD-HD and in 157 controls: without AF (NAF) and with ESKD-HD. The distribution of the risk alleles was compared in both groups and between different sub-phenotypes. The multilocus genetic risk score (GRS) was calculated to estimate the cumulative risk conferred by all SNPs. Several loci showed a trend toward an association with permanent AF (perm-AF): *CAV1*, *Cx40* and *PITX2*. However, GRS was significantly higher in the AF and perm-AF groups, as compared to NAF. Three of the tested variables were independently associated with AF: male sex, history of myocardial infarction (MI) and GRS. The GRS, which combined 13 previously described SNPs, showed a significant and independent association with AF in a Polish population of patients with ESKD-HD and concomitant AF. Further studies on larger groups of patients are needed to confirm the associations.

## Introduction

Atrial fibrillation (AF) is the most common heart rhythm disorder, and it is associated with increased morbidity and mortality and higher costs for the health care system, mainly due to thromboembolic complications^1,2^. AF affects about 6% of the population over the age of 65, and its prevalence increases with age^3^. According to epidemiological estimations, by the middle of this century, the number of patients with AF will double, and an AF episode will affect approximately 20–25% of people at some point in their lives^4,5^. The prevalence of AF is significantly higher in haemodialysed (HD) patients with end-stage kidney disease (ESKD) (ESKD-HD), and according to different data, between 15% and 30% of patients with ESKD-HD are affected^6^. In addition to classic AF risk factors, such as age, height, male sex, hypertension, diabetes, organic heart disease (myocardial infarction, heart failure and heart valve diseases), obesity and smoking, ESKD-HD patients exhibit new characteristic factors, including overhydration, anuria, hemodynamic instability during haemodialysis and ionic disorders in respect to kalemia, calcemia and magnesemia^6^. In addition, chronic kidney disease (CKD) itself is one of the strongest risk factors for cardiovascular diseases and cardiac arrhythmias; therefore, these diseases in CKD patients develop faster, and their symptoms and complications can be more severe.

The disease occurs in the form of the so-called ‘lone AF’ in 10–20% of all patients with AF, that is, despite the absence of the aforementioned risk factors^7^. Lone AF is a strictly electrophysiological disorder caused by disturbances in the flow of ionic currents on the surface of cardiomyocytes and between them^8^. Pathomechanism of AF becomes more complex, and it is not fully understood when the risk factors occur. It is suggested that the pathogenesis in this case involves the heterogeneous interactions of many factors, triggering mechanisms that cause rapid focal discharges and heterogeneous conduction of fibrillation type^9,10^.

Genetic factors play a key role in the pathogenesis of both ‘lone’ and ‘heterogenous’ AF^11–13^. The risk of lone AF was found to increase rapidly with the number of relatives with AF and with an early disease onset in these relatives^14^. Previous studies showed that the genetic background of lone AF involves mostly rare mutations of genes encoding components for potassium and sodium channels, but also for nuclear membrane structures, cardiac transcription factors and cardiomyocyte intercellular structures^15^. For the more frequent forms of ‘heterogeneous’ AF, a study of over 5,000 AF patients found that the risk of AF in patients with one parent with AF was more than four times greater than that of patients whose parents did not have cardiac arrhythmias^13^. The risk remained elevated even after the adjustement for classical AF risk factors. Recently, genomic-wide association studies (GWAS) have provided data indicating the involvement of commonly occurring single-nucleotide polymorphisms (SNPs) in the genetic predisposition to ‘heterogeneous’ AF^16–18^.

Although many of these loci have been identified, their individual effect on AF risk is modest^19^. For this reason, a genetic tool allowing for the assessment of the cumulative effect of multiple genetic markers on disease risk has recently been developed, what is called a genetic risk score (GRS)^20–22^. Previous studies showed that the combined assessment of multiple markers with low individual impacts provided a better risk estimation, especially for complex diseases, such as myocardial infarction, stroke, diabetes or rheumatoid arthritis^23–28^. Tada *et al*., when analysing the Swedish population, reported a GRS comprising 12 SNPs that conferred a two-fold increase in the risk of AF for patients grouped in the highest quintile of GRS compared to the lowest quintile. Moreover, the addition of this GRS to classical risk factors resulted in the classification of patients to other AF risk groups^29^. In another study on a population of American women, Everett *et al*. showed that GRS based on slightly different SNPs caused a 2.25-fold increase in AF risk, when compared the highest to the lowest GRS quintile, but adding this GRS to established risk factors did not alter the classification of patients’ 10-year disease risk assessment^30^. In a recent study, Lubitz *et al*. evaluated several different GRSs based on five population studies and found that a GRS comprising 11 SNPs showed a 1.4-fold increase in AF risk, when compared the highest to the lowest GRS quartile, while another one consisting of 25 different SNPs showed a maximum increase of 1.6-fold (also comparison of the highest to the lowest GRS quartile)^31^. However, such studies in CKD patients are still lacking.

The aim of the present study was to determine the association of 16 selected SNPs with AF risk in haemodialyzed patients with end-stage kidney disease using a large Polish population in the Mazovian region. We also wanted to evaluate the cumulative effect of all genotyped SNPs on AF risk and to identify the clinical and genetic factors that are independently associated with the risk of AF in this group of patients.

## Results

The demographic and clinical characteristics of the patients are presented in Table 1. The study and control groups differed significantly with respect to smoking habits and sex distribution.

**Table 1:** Study and control group characteristics.

	AF, n = 113	NAF, n = 157	*P*
Age, years	70.79 (10.34)	69.41 (8.99)	0.25
Males	74 (65.49%)	73 (46.50%)	0.002
BMI, kg/m^2^	26.28 (5.25)	28.87 (15.46)	0.1
Ever-smokers^a^	35 (59.32%)	46 (38.33%)	0.008
Duration of pre-ESKD period^b^, years	6.2 (6.21)	5.47 (5.52)	0.35
Presence of diuresis^c^	76 (79.17%)	129 (86%)	0.16
Uvol, mL^c^	693.23 (697.84)	569.87 (504.18)	0.11
Uvol ≥500 mL	60 (62.5%)	85 (56.67%)	0.36
Uvol 500–1000 mL	24 (25%)	47 (31.33%)	0.28
Uvol 1000–2000 mL	30 (31.25%)	33 (22%)	0.1
Uvol >2000 mL	6 (6.25%)	5 (3.33%)	0.28
Type of atrial fibrillation^d^			
-non-permanent	57 (51.35%)	N/A	
-permanent	54 (48.65%)		
Hypertension^e^	102 (96.23%)	149 (96.13%)	0.97
Coronary artery disease^f^	68 (64.76%)	87 (60%)	0.44
History of myocardial infarction^g^	35 (33.65%)	40 (28.57%)	0.39
Myocardial hypertrophy^h^	71 (77.17%)	104 (73.24%)	0.5

Data are presented as the mean (standard deviation) for continuous variables and number (percentage) for categorical variables. AF: study group (patients with atrial fibrillation). NAF: control group (patients without atrial fibrillation). ESKD: end-stage kidney disease. Uvol: 24-hour urine volume.

^a^Data available for 59 AF patients and 120 NAF patients.

^b^Time from the beginning of CKD to end-stage kidney disease.

^c^Data available for 96 AF patients and 150 NAF patients.

^d^Data available for 111 AF patients.

^e^Data available for 106 AF patients and 155 NAF patients.

^f^Data available for 105 AF patients and 145 NAF patients.

^g^Data available for 104 AF patients and 140 NAF patients.

^h^Data available for 92 AF patients and 142 NAF patients.

A full list of the analysed SNPs are presented in the Supplementary Table 1.

The SNPs’ association with AF are presented in Table 2. None of the individual analysed SNP’s showed the significant association with AF.

**Table 2:** SNPs’ associations with atrial fibrillation.

SNP	Gene	RA	N of cases AF/NAF	RAF AF/NAF	OR (95% CI)	*P*_*uncorr*_
rs1805127	*KCNE1*	C	102/138	0.67/0.67	1.01 (0.69–1.49)	0.92
rs3807989	*CAV1*	G	98/132	0.64/0.55	1.45 (0.99–2.12)	0.054
rs2106261	*ZFHX3*	T	99/134	0.20/0.18	1.16 (0.73–1.85)	0.53
rs2200733	*PITX2*	T	100/132	0.19/0.14	1.39 (0.85–2.3)	0.19
rs3853445	*PITX2*	T	98/132	0.72/0.72	0.99 (0.66–1.50)	1
rs13376333	*KCNN3*	T	101/137	0.32/0.29	1.15 (0.78–1.71)	0.48
rs1805123	*KCNH2*	T	98/139	0.81/0.82	0.91 (0.57–1.46)	0.71
rs4845625	*IL6R*	T	101/139	0.47/0.50	0.88 (0.61–1.27)	0.50
rs11047543	*SOX5*	G	100/137	0.88/0.88	0.99 (0.56–1.73)	1
rs10465885	*Cx40*	T	98/132	0.53/0.44	1.43 (0.98–2.09)	0.06
rs13038095	*SULF2*	T	101/132	0.13/0.10	1.36 (0.76–2.42)	0.3
rs6800541	*SCN10A*	T	102/137	0.63/0.66	0.89 (0.54–1.49)	0.67
rs251253	*NKX2-5*	T	104/133	0.61/0.57	1.18 (0.81–1.70)	0.39

AF: atrial fibrillation group. CI: confidence interval. NAF: non- atrial fibrillation group. OR: odds ratio. *P*_*uncorr*_: uncorrected *P* value. RA: risk allele. RAF_AF_: RA frequency in AF group. RAF_NAF_: RA frequency in NAF group. SNP: single-nucleotide polymorphism.

The analysis of the sub-groups showed a trend toward association (nominal association – *P* < 0.05, no association after Bonferroni correction – *P* > 0.05) of three SNPs – rs3807989, rs2200733, rs10465885 – with permanent AF (Table 3).

**Table 3:** SNPs’ associations with non-permanent (NP-AF) and permanent (P-AF) atrial fibrillation. A Bonferroni corrected P value is provided for nominal (uncorrected *P* value < 0.05) associations.

SNP	NP-AF			P-AF			
	RAF	OR (95% CI)	*P*_*uncorr*_	RAF	OR (95% CI)	*P*_*uncorr*_	*P*_*corr*_
rs1805127	0.66	0.96 (0.61–1.59)	1	0.676	1.05 (0.64–1.70)	0.86	
rs3807989	0.62	1.29 (0.80–2.07)	0.30	0.670	1.64 (1.01–2.65)	**0.044**	0.572
rs2106261	0.21	1.25 (0.70–2.28)	0.45	0.190	1.08 (0.60–1.94)	0.81	
rs2200733	0.15	1.01 (0.52–1.97)	1	0.235	1.82 (1.01–3.25)	**0.043**	0.559
rs3853445	0.74	1.12 (0.66–1.90)	0.68	0.694	0.89 (0.54–1.48)	0.66	
rs13376333	0.30	1.04 (0.63–1.71)	0.89	0.343	1.27 (0.78–2.06)	0.34	
rs1805123	0.78	0.76 (0.43–1.33)	0.34	0.833	1.07 (0.58–1.99)	0.82	
rs4845625	0.48	0.94 (0.59–1.48)	0.78	0.451	0.83 (0.53–1.31)	0.43	
rs11047543	0.86	0.87 (0.45–1.70)	0.68	0.890	1.15 (0.56–2.36)	0.71	
rs10465885	0.46	1.09 (0.68–1.76)	0.72	0.602	1.96 (1.22–3.15)	**0.005**	0.065
rs13038095	0.12	1.16 (0.55–2.46)	0.69	0.150	1.55 (0.78–3.06)	0.21	
rs6800541	0.67	1.03 (0.63–1.66)	0.92	0.598	0.76 (0.48–1.22)	0.26	
rs251253	0.61	1.15 (0.73–1.83)	0.55	0.615	1.20 (0.75–1.91)	0.44	

CI: confidence interval. NP-AF: non-permanent AF. PAF: permanent AF. OR: odds ratio. *P*_*corr*_: corrected *P* value (Bonferroni correction). *P*_*uncorr*_: uncorrected *P* value. RAF: risk allele frequency. SNPs: single-nucleotide polymorphisms. *P* < 0.05 are in bold.

To assess the cumulative risk conferred by multiple loci, the GRS was calculated (Table 4). An example of the calculation of GRS was placed in the Supplementary Table 2. The GRS was significantly higher in the AF group and perm-AF group compared to the NAF group.

**Table 4:** GRS in controls (NAF), AF patients (AF), AF patients with non-permanent AF (NP-AF) and AF patients with permanent AF (P-AF).

	GRS, mean (SD)	P*
NAF (n = 132)	0.688 (0.245)	n/a
AF (n = 98)	0.761 (0.280)	**0.036**
NP-AF (n = 48)	0.711 (0.257)	0.591
PAF (n = 50)	0.810 (0.294)	**0.005**

*Versus controls. *P* < 0.05 are in bold.

A logistic regression with nine variables (age, sex, smoking, BMI, presence of diuresis, coronary artery disease, myocardial infarction, myocardial hypertrophy and GRS) showed that three are significantly associated with AF: male sex, myocardial infarction and GRS (Table 5). It should be emphasised that OR for GRS in Table 5 is a 0.1 unit odds ratio (i.e. an OR of 1.24 is a ratio for a 0.1-unit increase in GRS). The model including these three variables (i.e. male sex, myocardial infarction and GRS) was found to cover 9.3% of phenotypic variation, and the post-hoc analysis showed that it had a correct classification of 75% of the cases.

**Table 5:** Variables associated with AF.

Variable	OR (95% CI)	*P*
Male sex	2.32 (1.13–4.78)	0.023
Myocardial infarction	2.66 (1.23–5.75)	0.013
GRS	1.24 (1.07–1.43)^#^	0.005

CI: confidence interval; OR: odds ratio; GRS: genetic risk score;

^#^The 0.1 unit odds ratio, i.e. the ratio for a 0.1-unit change in the predictor (GRS).

## Discussion

To the best of our knowledge, this is the first report that focused on the genetic background of AF in haemodialysed patients with ESKD. The weighted GRS comprising 13 SNPs (hereafter called Saracyn-GRS), was significantly higher in the AF group and perm-AF group as compared to the control group (NAF). In addition, GRS along with two other variables (i.e. male sex and history of MI) showed an independent association with AF. The disease model containing these three variables covered nearly 10% of the phenotypic variability, and the post-hoc analysis showed that it gave the proper classification for 75% of these cases.

Several studies have focused on the genetics of AF in the general population. However, the literature on genetic risk scores for AF is still scarce and non-existent for ESKD-HD patients. Tada *et al*. for instance, using a prospective observational study for a Swedish population (The Malmö Diet and Cancer study), showed that GRS composed of 12 SNPs (hereafter called Tada-GRS) was associated with at least one AF incident throughout 14 years of follow-up, even after adjusting for non-genetic AF risk factors such as age or hypertension^29^. Patients grouped in the highest quintile of Tada-GRS had a nearly two-fold higher risk of AF than those in the lowest quintile. Furthermore, the effect of Tada-GRS, as an independent AF risk factor, was comparable to one of the strongest classical AF risk factors: hypertension. Patients with high Tada-GRS and without hypertension had a similar risk of AF as those with hypertension and low Tada-GRS. The comparable effect of Tada-GRS and hypertension was observed in both groups below and over 57 years of age. Our work is difficult to compare to Tada *et al*.’s study for several reasons. The work of Tada *et al*. is a large observational study conducted on the Malmö local community, though age, sex, BMI, and smoking rates are similar. However, because of the nature of the underlying disease – ESKD – our group of patients is different, with significantly higher rates of hypertensive patients, coronary artery disease (CAD), cardiac hypertrophy or history of myocardial infarction. Moreover, there were only five common SNPs between the Tada-GRS and our GRS. In addition, in the study by Tada *et al*., there were 10 SNPs nominally associated with AF, possibly due to the large population of patients. Furthermore, in contrast to the study by Tada *et al*., in our study, hypertension was not significantly associated with AF. Probably, because of the nature of ESKD, it was very common in all groups of patients both with and without the AF. Hence, the fundamental differences between these two studies result from different groups of patients and, in fact, from the presence of ESKD and its consequences; however, Tada *et al*. and the current study demonstrated the utility of GRS in estimating the risk of AF.

In another study on a large European population, Lubitz *et al*. showed that multi-allelic GRS (hereafter called Lubitz-GRS) combining 12 SNPs that were potentially associated with AF (each SNP was nominally associated with AF risk) and previously confirmed in the genome-wide association studies (GWAS) was associated with a five-fold increase in the risk of AF in the group of patients with the highest number of risk alleles compared to patients with the lowest number of such alleles^32^. Moreover, the study was successfully replicated in a population of Japanese patients with AF; except in this case, the increase of risk associated with AF was four-fold. In both populations, the risk gradually increased as the number of AF risk alleles rose, with the most numerous population (nearly 25%) of patients having an average (9–10) number of AF risk alleles. Furthermore, none of the analysed SNPs were significantly associated either with the survival or the mortality of the patients. It is also hard to compare the study by Lubitz *et al*. with the current study, mainly because of fundamentally different populations, the underlying disease in our study (i.e., ESKD) and the high heterogeneity of patients’ clinical data in the study by Lubitz *et al*. The components of both GRSs (Lubitz-GRS vs. Saracyn-GRS) were also different because they overlapped in two of the total of 12 and 13 SNPs used for the analysis in Lubitz *et al*. and in the current work, respectively. Therefore, Lubitz *et al*. and the current study have demonstrated the usefulness of GRS in AF risk assessment; however, in Lubitz *et al*. it was not surprising because almost all their SNPs were nominally and independently associated with AF. Our GRS, with lacking such unequivocal associations for single SNPs, has brought a new value.

In another publication, Everett *et al*. studied a large prospective cohort study of American women of European descent (part of the Women’s Health Study [WHS]) with a follow-up period of over 12 years. The study showed that GRS (hereafter called Everett-GRS) consisting of 12 SNPs potentially associated with AF increased the risk of AF 2.25-fold in the group of patients from the highest quintile of GRS compared to the lowest quintile^30^. However, the study also demonstrated that the addition of this GRS to pre-established clinical 10-year risk categories of AF did not alter the classification of these patients. Everett *et al*.’s study and the current one are difficult to compare for several reasons. Most of all, the current study involves a different population, that is haemodialysed patients with ESKD. Moreover, the study by Everett *et al*. included exclusively women, while the male gender is a strong independent AF risk factor. Additionally, these women were without CVD and heart failure (HF), additional AF risk factors. Finally, our GRS and Everett-GRS had only four common SNPs. In addition, of the 12 SNPs selected by Everett *et al*., five were nominally associated with AF, which undoubtedly affected the overall GRS estimation. Only seven of the 12 SNPs in the study by Everett *et al*. were directly genotyped, while the remaining were imputed. In our study, we genotyped all selected SNPs, of which 13 were used in the final analysis.

In the most recently published research, Lubitz *et al*. once again focused on the issue of GRS and its role in AF risk assessment but used a slightly different material and method^31^. The authors combined the data from five different prospective studies with a 5-year follow-up and found that the GRS combining 25 SNPs (hereafter named as Lubitz-GRS 2) is associated with a 67% increase in the risk of AF when comparing the highest quartile of the GRS to the lowest one. All SNPs used to calculate this GRS were nominally associated with AF. Moreover, the addition of this GRS to the classical AF risk factors in the discriminatory model changed its parameters (C-statistics) only minimally; additionally, it significantly differed between the five analysed cohorts. To compare this work with the current study, we would have to compare our material with the five different cohorts used by Lubitz *et al*. Our GRS, consisting of 13 SNPs, was associated with a significant increase of AF risk in the group of patients with ESKD-HD, but only with three of 13 SNPs trending toward association with perm-AF.

Of the 13 SNPs that finally qualified for the analysis in our study, three showed a trend toward an association with permanent AF. However, it should be emphasised that none of the SNPs withstood the Bonferroni correction; thus, further studies on larger groups of patients are needed to confirm these associations. Interestingly, the SNPs showing a trend toward an association with perm-AF (rs3807989, rs10465885 and rs2200733) lie within the genes that encode proteins of atrial anatomic structures or proteins involved in their morphogenesis, that is, *CAV1*, *Cx40* and *PITX2*, respectively^33,34^.

Caveolin-1 (CAV1) is a basic component of the characteristic cell membrane structure called caveolae^35^. It also actively interacts with the different type of the potassium channels, influences the transforming growth factor-beta 1 (TGF-β1) signalling pathway and regulates the intracellular nitric oxide pathway, disorders of which are one of the pathogenetic mechanisms behind AF^36–39^. Cell signalling defects as mentioned above can encounter additional mechanisms of arrhythmias in patients with ESKD/HD^40^. During 4 hours of HD, kalemia is able to decrease by approximately 2.5–3.5 mmol/L, on average. Such a difference in potassium concentration in patients without other significant complications usually does not cause a rhythm disorder. However, a coexistence of ultrastructural CAV1-dependent defects of cardiomyocytes, which can disturb the normal potassium current and serious changes in potassium concentration during HD sessions, can lead to amplification of strong pathomechanisms of arrhythmias.

Connexin, in turn, is the main component of the gap junctions^41–44^. Changes in the number of gap junctions on the cell’s surface and their distribution (lateralisation), as well as structural changes, may result in disturbances of the flow of different signals and the development of arrhythmias^45^. Another critical factor of rhythm disorders in ESKD/HD patients can be a fluctuation of calcemia. Calcium regulates intercellular cross-talk within the gap junction and physiological function of enzymes and cell proteins^46^. During a 4-hour HD session, calcium concentration can decrease by as much as 0.5–1.0 mmol/L (i.e. 50% of the initial blood concentration). Coexistence of ultrastructural defects of intercellular junctions, which control calcium flow and HD-dependent changes of calcium concentration, can lead to serious disorder of calcium currents and development of a heart rhythm disorder.

Finally, a paired-like transcription factor (PITX2) is involved in asymmetric atrial morphogenesis^47–49^. It also influences the proper functioning of ion channels, gap and tight junctions in the cardiomyocyte cell membrane^50^. Recently, it has also been found that PITX2c expression in cardiomyocytes in patients with perm-AF is significantly reduced, confirming the existence of a molecular mechanism linking PITX2 dysfunction with AF development. Patients with ESKD can suffer from chronic overhydration between HDs and from rapid dehydration (even 4.0–5.0 L) during an HD session^51^. These extreme changes of volemia usually provoke rapid blood pressure drops, incidents of tachycardia during HD and, with the coexistence of PITX2-dependent atrium structure and cell membrane defects can also lead to accumulation of these two arrythmogenic mechanisms and generate a fleet development of atrial fibrillation.

In ESKD-HD patients, these morphogenetic mechanisms appear to be an ‘ideal’ backgrounds for AF development. Based on these genetic predispositions, existing ESKD and renal replacement therapy would include additional risk factors for AF, such as chronic overhydration, rapid dehydration, rapid blood pressure drops, tachycardia episodes and/or ionic disorders, during haemodialysis^6^.

As a strength of the current work, it should be emphasised that our study is the first concerning the genetic background of AF in patients with ESKD-HD. Therefore, it is difficult to compare our results with those from previous studies conducted on different populations. The GRS constructed for this study was significantly higher in AF and perm-AF patients compared to the controls. It was also significantly and independently from other risk factors associated with AF. The GRS also differentiated between patients with permanent and non-permanent AF (i.e. help identify patients at risk of disease chronification and patients with a lower chance of heart rhythm normalisation). Although we have found no associations with AF for single SNPs, we have shown a positive association with multiple genetic marker. This new genetic tool, which is composed of 13 previously described SNPs, has been applied for the assessment of AF risk in ESKD/HD patients for the first time. The assessment of cardiac arrhythmias risk during HD can be a critical condition and practical tool for an adequate and safe haemodialysis sessions.

A few limitations of the present work should also be noted. The study group, even though involving haemodialysed ESKD patients from the 5.5 million population in the Mazovian region (approximately one-sixth of the population and area of Poland), was relatively small; therefore, our study had limited power in detecting associations of single SNPs with AF. However, to overcome this problem, we used a combined weighted GRS as the main genetic variable. The constructed 13-SNP GRS was significantly associated with AF independent from other risk factors. In addition, the study and control groups differed in sex proportions and percentage of ever smokers. However, these parameters reflect the clinical data and the clinical distribution of AF risk factors, and we recruited all consecutive patients with AF and ESKD/HD from the Mazovian region.

In summary, although we observed only a trend toward an association of several previously reported SNPs with permanent AF in ESKD-HD patients, the multiple loci genetic risk score, composed of 13 SNPs, was significantly higher in the AF group and permanent AF group compared to the controls. Moreover, the association of GRS with AF was independent of classical risk factors. The disease model comprising three variables (i.e. GRS, age and myocardial infarction) covered nearly 10% of the phenotypic variability, and the post-hoc analysis demonstrated that it allowed for a proper classification of 75% of the cases. This new genetic tool, composed of 13 previously described SNPs, has been applied for the assessment of AF risk in ESKD/HD patients for the first time in literature. The assessment of cardiac arrhythmias risk during HD can be a critical condition and practical tool for an adequate and safe haemodialysis sessions.

## Material and Methods

The study was approved by the Military Institute of Medicine Ethics Committee. Informed consent was obtained from each patient. All procedures were performed in accordance with the Helsinki Declaration of 1975, revised in 1983.

### Patients and controls

Initially 115 consecutive AF-HD patients from the group of 1126 ESKD/HD patients were included in the study; however, 2 patients were excluded from the study because of the poor DNA quality. Finally, the study group comprised 113 haemodialysed patients with ESKD and a history of AF. The patients were recruited from the Military Institute of Medicine in Warsaw and Mazovian Centers of Dialysis in Radom, Ciechanów, Grodzisk Mazowiecki, Maków Mazowiecki, Sokołów Podlaski, Skierniewice, Warszawa Międzylesie, Wołomin and Otwock. The inclusion criteria were as follows: (i) ESKD treated by haemodialysis, (ii) presence or history of AF (confirmed by electrocardiography) and (iii) age ≥18 years. The exclusion criteria included the following: (i) congenital heart defect, (ii) history of heart surgery and (iii) history of malignancy. The AF patients were classified as having non-permanent AF (nperm-AF), defined as paroxysmal or persistent (lasting less than 12 months) AF, or permanent AF (perm-AF), defined as AF lasting over 12 months. One hundred and seventy two age-matched controls were enrolled in the study (cases to controls ratio 2:3). However, 5 controls were excluded from the study because of the poor DNA quality, and further 10 controls were excluded because of incorrect enrollment (subjects not fulfilling inclusion/exclusion criteria- mostly concomitant heart arrhythmias other than AF). Finally, the control group consisted of 157 patients with ESKD (non-atrial fibrillation group: NAF group). The inclusion criteria were as follows: (i) ESKD treated by haemodialysis and (ii) age ≥18 years. The exclusion criteria included the following: (i) history or presence of AF, (ii) history of cardiac arrhythmia other than AF and (iii) history of cardiac ablation. A structured questionnaire was used to collect data regarding age, gender, smoking habits, body mass index (BMI), duration of pre-ESKD period, presence and volume of diuresis, coexistence of hypertension or coronary artery disease, history of myocardial infarction and presence of myocardial hypertrophy (confirmed by echocardiography).

### Single-nucleotide polymorphisms selection

SNPs previously associated with AF were selected for this study^52–62^. The criteria for SNP selection were the following: (i) association with AF confirmed in GWAS, a meta-analysis or large-scale case-control study and (ii) minor allele frequency (MAF) ≥0.05 in a Caucasian population (based on data from the HapMap CEU population). Using these criteria, 16 SNPs were selected for genotyping. However, three of the genotyped SNPs were excluded from the analysis because of low genotyping success rate (<80%) or Hardy-Weinberg equilibrium deviation: rs3825214 (*TBX5*), rs1131820 (*KCNN3*) and rs11708996 (*SCN5A*). The complete list of the analysed SNPs is presented in Table S1.

### Genotyping

DNA was isolated from whole-blood samples using a salting-out method^63^. For SNPs genotyping a custom array was designed (Taqman OpenArray Genotyping Plate, Custom Format 16 QuantStudio 12 K Flex, Life Technologies, Carlsbad, CA, USA) and genotyping was performed according to the manufacturer’s protocols on a QuantStudio 12 K Flex Real-Time PCR System (Applied Biosystems, Foster City, CA, USA).

### Statistical analysis

The PLINK statistical software package was used to test the Hardy-Weinberg equilibrium (HWE) and to assess the differences in allele frequencies of each SNP between the cases and controls^64^. *P* < 0.05 was considered a significant deviation from the HWE. The remaining statistical analyses were performed using Statistica 12 package (StatSoft Inc). To correct for multiple testing, we applied a Bonferroni correction (*P* value multiplied by the number of the analysed SNPs). The GRS was calculated to assess the cumulative risk conferred by multiple loci. GRS was computed as the number of risk alleles multiplied by the natural logarithm of the odds ratio (OR) associated with each individual SNP. ORs from previous studies (Table S1) were used to calculate the GRS. Because of missing data, 15 AF patients and 25 NAF patients were excluded from the GRS analysis. The GRS in the study and control groups was compared with a student’s t test. A logistic regression was used to predict the factors associated with AF and calculate the phenotypic variation covered by the model (McFadden R^2^ for logistic regression). The R^2^ was defined as R^2^ = 1 − ln(L_m_)/ln(L_0_), where L_0_ is the value of the likelihood function for a model with no predictors and L_m_ is the likelihood for the model being estimated.

### Data availability

All data generated and analysed during the current study are available from the corresponding author on reasonable request.

## Electronic supplementary material

Supplementary Table 1 and 2
