# Supplementary material for: Value of multilocus genetic risk score for atrial fibrillation in end-stage kidney disease patients in a Polish population
Source: Sci Rep. 2018 Jun 18;8:9284. doi: 10.1038/s41598-018-27382-5 (PMC6006310; doi:10.1038/s41598-018-27382-5)
Supplement: Supplementary file 1 — Supplementary Table 1 and 2 [file 41598_2018_27382_MOESM1_ESM.docx]

*Manuscript number: SREP-17-50594B*

Title:

Value of multilocus genetic risk score for atrial fibrillation in end-stage kidney disease patients in a Polish population

Authors:

Marek Saracyn, Bartłomiej Kisiel, Artur Bachta, Maria Franaszczyk, Dorota Brodowska-Kania, Wawrzyniec Żmudzki, Konrad Szymański, Antoni Sokalski, Wiesław Klatko, Marek Stopiński, Janusz Grochowski, Marek Papliński, Zdzisław Goździk, Longin Niemczyk, Barbara Bober, Maciej Kołodziej, Witold Tłustochowicz, Grzegorz Kamiński, Rafał Płoski, Stanisław Niemczyk

Supplementary Table 1 and 2 have been placed below.

**Supplementary Table 1. A full list of the analyzed SNPs.**

| **SNP** | **Gene** | **Chromosomal localization** | **Reference** | **Effect allele*** | **OR (95% CI)*** | ***P**** | **Cases*** | **Controls*** |
| --- | --- | --- | --- | --- | --- | --- | --- | --- |
| **rs1805127** | *KCNE1* | 21q22.12 | 1 | C | 1.53 (1.41-1.67) | <0.0001 | 2,099 | 2,252 |
| **rs3807989** | *CAV1* | 7q31 | 2 | A | 0.90 (0.87-0.92) | 3.6 x 10^-12^ | 12,088 | 62,456 |
| **rs2106261** | *ZFHX3* | 16q22 | 3 | T | 1.21 (1.13-1.29) | 1 x 10^-8^ | 12,694 | 132,602 |
| **rs2200733** | *PITX2* | 4q25 | 3 | T | 1.67 (1.50-1.86) | 2 x 10^-21^ | 12,694 | 132,602 |
| **rs3853445** | *PITX2* | 4q25 | 4 | C | 0.71 (0.61-0.84) | 4.1 x 10^-5^ | 790 | 1,177 |
| **rs13376333** | *KCNN3* | 1q21 | 5 | T | 1.56 (1.38-1.77) | 6.3 x 10^-12^ | 1,335 | 12,844 |
| **rs1805123** | *KCNH2* | 7q36.1 | 6 | T | 1.25 (1.11-1.41) | 0.00033 | 1,207 | 2,475 |
| **rs4845625** | *IL6R* | 1q21.3 | 8 | C | 0.90 (0.85-0.95) | 0.0005 | 2,260 | 16,264 |
| **rs11047543** | *SOX5* | 12p12 | 9 | G | 1.13 (1.06-1.20) | 2.1 x 10^-4^ | 5,741 | 41,342 |
| **rs10465885** | *Cx40* | 1q21.1 | 11 | A | 1.30 (1.07-1.58) | 0.011 | 342 | 534 |
| **rs13038095** | *SULF2* | 20q13.3 | 5 | T | 1.61 (1.37-1.91) | 1.1 x 10^-8^ | 1,335 | 12,844 |
| **rs6800541** | *SCN10A* | 3p22.2 | 9 | C | 0.92 (0.88-0.96) | 1.5 x 10^-4^ | 5,741 | 41,342 |
| **rs251253** | *NKX2-5* | 5q35 | 9 | T | 1.07 (1.03-1.12) | 1.3 x 10^-3^ | 5,741 | 41,342 |

CI- confidence interval; OR- odds ratio; *data from the literature.

**Supplementary Table 2. An example of GRS calculation.**

| **SNP** | **Effect allele*** | **OR*** | **logOR** | **Patient #1** | | **Patient #2** | |
| --- | --- | --- | --- | --- | --- | --- | --- |
|  |  |  |  | **genotype** | **logOR*EAN** | **genotype** | **logOR*EAN** |
| **rs1805127** | C | 1.53 | 0,184691431 | C/C | 0,369382862 | T/T | 0 |
| **rs3807989** | A | 0.90 | -0,045757491 | A/A | -0,09151498 | A/A | -0,09151498 |
| **rs2106261** | T | 1.21 | 0,082785 | C/C | 0 | C/C | 0 |
| **rs2200733** | T | 1.67 | 0,222716 | C/C | 0 | C/C | 0 |
| **rs3853445** | C | 0.71 | -0,14874 | T/T | 0 | C/T | -0,14874 |
| **rs13376333** | T | 1.56 | 0,193125 | C/T | 0,193125 | C/C | 0 |
| **rs1805123** | T | 1.25 | 0,09691 | T/T | 0,193820026 | G/T | 0,09691 |
| **rs4845625** | C | 0.90 | -0,04576 | C/T | -0,04576 | T/T | 0 |
| **rs11047543** | G | 1.13 | 0,053078 | G/G | 0,106156886 | G/G | 0,106156886 |
| **rs10465885** | A | 1.30 | 0,113943 | G/G | 0 | G/A | 0,113943 |
| **rs13038095** | T | 1.61 | 0,206826 | G/G | 0 | G/G | 0 |
| **rs6800541** | C | 0.92 | -0,03621 | C/C | -0,07242 | T/T | 0 |
| **rs251253** | T | 1.07 | 0,029384 | C/C | 0 | T/T | 0,058767556 |
| **GRS: Σ(logOR*EAN)** |  |  |  |  | 0,652788 |  | 0,135521 |

OR- odds ratio; EAN- effect alleles number *data from the literature
